# TAVR and cancer: machine learning-augmented propensity score mortality and cost analysis in over 30 million patients

**DOI:** 10.1186/s40959-021-00111-0

**Published:** 2021-06-28

**Authors:** Dominique J. Monlezun, Logan Hostetter, Prakash Balan, Nicolas Palaskas, Juan Lopez-Mattei, Mehmet Cilingiroglu, Zaza Iakobishvili, Michael Ewer, Konstantinos Marmagkiolis, Cezar Iliescu

**Affiliations:** 1 Department of Cardiology, The University of Texas M.D. Anderson Cancer Center, 1400 Pressler Street, Unit 1451, Houston, TX 77030 USA; 2 Global System Analytics & Structures, New Orleans, USA; 3 Division of Cardiology, The University of Texas Health Sciences Center at Houston, Houston, TX USA; 4 Division of Cardiovascular Disease, University of Hawaii John Burns School of Medicine, Honolulu, HI USA; 5 Department of Community Cardiology, Tel Aviv University, Tel Aviv, Israel

**Keywords:** TAVR, Cancer, Mortality, Disparities

## Abstract

**Introduction:**

Cardiovascular disease (CVD) and cancer are the top mortality causes globally, yet little is known about how the diagnosis of cancer affects treatment options in patients with hemodynamically compromising aortic stenosis (AS). Patients with cancer often are excluded from aortic valve replacement (AVR) trials including trials with transcatheter AVR (TAVR) and surgical AVR (SAVR). This study looks at how cancer may influence treatment options and assesses the outcome of patients with cancer who undergo SAVR or TAVR intervention. Additionally, we sought to quantitate and compare both clinical and cost outcomes for patients with and without cancer.

**Methods:**

This population-based case-control study uses the most recent year available National Inpatient Sample (NIS (2016) from the United States Department of Health and Human Services’ Agency for Healthcare Research and Quality (AHRQ). Machine learning augmented propensity score adjusted multivariable regression was conducted based on the likelihood of undergoing TAVR versus medical management (MM) and TAVR versus SAVR with model optimization supported by backward propagation neural network machine learning.

**Results:**

Of the 30,195,722 total hospital admissions, 39,254 (0.13%) TAVRs were performed, with significantly fewer performed in patients with versus without cancer even in those of comparable age and mortality risk (23.82% versus 76.18%, *p* < 0.001) despite having similar hospital and procedural mortality. Multivariable regression in patients with cancer demonstrated that mortality was similar for TAVR, MM, and SAVR, though LOS and cost was significantly lower for TAVR versus MM and comparable for TAVR versus SAVR. Patients with prostate cancer constituted the largest primary cancer among TAVR patients including those with metastatic disease. There were no significant race or geographic disparities for TAVR mortality.

**Discussion:**

Comparison of aortic valve intervention in patients with and without cancer suggests that interventions are underutilized in the cancer population. This study suggests that patients with cancer including those with metastasis have similar inpatient outcomes to patients without cancer. Further, patients who have symptomatic AS and those with higher risk aortic valve disease should be offered the benefit of intervention. Modern techniques have reduced intervention-related adverse events, provided improved quality of life, and appear to be cost effective; these advantages should not necessarily be denied to patients with co-existing cancer.

## Introduction

Cardiovascular disease (CVD) and cancer remain the top causes of mortality in developed countries with their incidences continuing to rise with aging populations [1]. Aortic stenosis (AS), the CVD in which the heart’s aortic valve narrows, similarly has a growing prevalence but includes a mortality rate as high as 50% [2]. Though its age-adjusted mortality overall has fallen recently with the rise of transcatheter aortic valve repair (TAVR) particularly for patients with historically high surgical risk (which increases with age as does AS incidence) [2], AS mortality has remained stable for black, Hispanic, and rural patients. This is particularly concerning for racial and socioeconomic disparities in access to life-saving TAVR treatment. The above mortality and disparity challenges may be exacerbated by the co-prevalence of cancer, which also is increasingly discovered in older populations, though little is known about how AS outcomes and treatment in patients with cancer differs from those without cancer. Furthermore, patients with cancer are often excluded from aortic valve replacement (AVR) trials, making direct outcome comparisons problematic [3].

TAVR initially emerged as the main treatment option for patients with severe AS who are at high risk for surgery [4]. More recently, evidence extended the indication to intermediate and low-risk patients [5–7]. When compared with surgical aortic valve repair (SAVR), TAVR appears cost-effective for high-risk patients and offers considerable cost savings for the group of intermediate risk patients [8, 9]. Single center experience suggests that both SAVR [10] and transcatheter (TAVR) versus medical management (MM) improve survival with an incremental survival advantage of up to 36 months [11]. These trends underscore the increasing evidence that TAVR versus surgical AVR (SAVR) patients have comparable mortality, stroke, and rehospitalization rates at one and 2 years for high, intermediate and now low surgical risk patients [4]. Lower cost derived from reduced length of stay (LOS) in the index hospitalization as well as less costly follow-up surveillance translated in decreased lifetime costs by $8000–$10,000 while improving overall quality of live and quality-adjusted survival.

The number of TAVR patients has increased substantially, especially in older patients; the extent to which patients with cancer have benefitted from this change is less clear, but as older patients are more likely to be affected by cancer, one would expect that TAVR procedures would have a parallel increase in such patients. Initial non-randomized small sample studies showed comparable 30-day results and increased 1-year mortality in patients with cancer receiving TAVR, possibly related to progression of the underlying cancer or its treatment [12, 13].

The present study was therefore undertaken to compare patients with and without cancer undergoing TAVR and the clinical and cost outcomes of these patients relative to MM and SAVR. We further sought to estimate the extent of utilization of TAVR in the cancer population.

## Methods

### Study design

This is the first known nationally representative population-based case-control study comparing TAVR, SAVR, and MM clinical and cost outcomes among all hospitalized patients (with and without cancer and within individual primary cancers), and the first to apply a machine learning-augmented propensity score adjusted multivariable regression methodology for such a cardio-oncology study. It uses the recent 2016 NIS dataset to allow optimal generalizability to current practice. The data was available from The United States Department of Health and Human Services’ Agency for Healthcare Research and Quality (AHRQ). Study inclusion criteria were 2016 hospitalizations of adults 18 years of age or older with documented mortality and presence or absence of cancer. This study using de-identified data was performed in accordance with the Declaration of Helsinki and did not require Institutional Review Board approval. The National Inpatient Sample (NIS) discharge weights were utilized to calculate national estimates, allowing optimal generalizability to current practice.

Subjects undergoing TAVR specifically were identified by the ICD-10 procedure codes of 02RF37H (“Replace of Aort Valve with Autol Sub, Transap, Perc Approach”), 02RF37Z (“Replacement of Aortic Valve with Autol Sub, Perc Approach”), 02RF38H (“Replace Aort Valve w Zooplastic, Transap, Perc”), 02RF38Z (“Replacement of Aortic Valve with Zooplastic, Perc Approach”), 02RF3JZ (“Replacement of Aortic Valve with Synth Sub, Perc Approach”), 02RF3KH (“Replace Aort Valve w Nonaut Sub, Transap, Perc”), 02RF3KZ (“Replacement of Aortic Valve with Nonaut Sub, Perc Approach”), 02RF47Z (“Replacement of Aort Valve with Autol Sub, Perc Endo Approach”), 02RF48Z (“Replace of Aort Valve with Zooplastic, Perc Endo Approach”), 02RF4JZ (“Replacement of Aort Valve with Synth Sub, Perc Endo Approach”), and 02RF4KZ (“Replace of Aort Valve with Nonaut Sub, Perc Endo Approach”). MM was identified by necessity due to data limitations according to patients with AS who did not undergo TAVR or SAVR in the index study period.

ICD-10 codes were also used to identify demographics, comorbidities, outcomes, and malignancies. As detailed by the NIS and available on their dataset website, malignancies were identified using the above codes according to primary organ site (including brain and nervous system, head or neck, thyroid, breast, lung, esophagus, stomach, pancreas, liver or bile system, rectum or anus, colon, peritoneum, bone or connective tissue system, hematological malignancies [including Hodgkin lymphoma, Non-Hodgkin lymphoma, leukemia, and multiple myeloma], skin, uterus, cervix, ovarian, prostate, testes, bladder, and renal). Metastatic cancer was identified by the above coding system if a patient carried the additional classification of metastatic disease in addition to the primary organ site. Healthcare Cost and Utilization Project (HCUP) tools such as the Clinical Classification Software, which had been used prior to the NIS 2016 dataset for such purposes as classifying cancer (e.g., by primary type, current versus historical), were not used in this study because they were found by HCUP as a beta version to be unreliable when applied to the 2016 dataset’s ICD-10 data.

### Clinical outcomes

The clinical outcomes compared the cancer population with similar patients who did not have cancer. Secondary assessed outcomes included mortality, post-procedure pacemaker implantation, total direct cost, and outcome racial and geographic disparities. The associations assessed included these outcomes and the following predictors: TAVR versus MM and TAVR versus SAVR (additionally by cancer versus non-cancer among all hospitalized patients, and in sub-group analysis within patients with cancer [by metastatic versus non-metastatic malignancy, active versus prior cancer, solid versus hematological cancer,, and primary cancer types], patients with thrombocytopenia, and patients with prior radiation treatment particularly mediastinal). Propensity score matching was utilized to reduce bias and unequal distribution of treatment between patients with and without cancer, particularly in the assessment of TAVR versus MM (considering the absence of aortic stenosis severity documented in the data) given the clinical importance of this assessment, the paucity of data on this comparison in cancer versus without cancer, and the typical exclusion of patients with cancer from TAVR and SAVR randomized trials (and thus in current medical practice a possible tendency away from providing non-medical intervention for patients with cancer leaving the majority of such patients to be managed medically).

### Statistical analysis

Descriptive statistics and bivariable analysis by mortality were performed for the overall sample. Independent sample t-test was conducted to assess means and Wilcoxon rank sum tests for medians for continuous variables. Pearson’s chi square test or Fisher’s exact test were conducted to assess proportions for categorical variables. Multivariable regression was then conducted for the primary outcome of in-patient mortality and the secondary outcomes of length of stay (LOS, in days) and total direct cost (also adjusting for LOS); sub-group analysis within TAVR subjects of the above outcomes were also conducted to assess possible race and geographic disparities. Propensity scores based on the likelihood of undergoing TAVR versus MM and TAVR versus SAVR were also calculated and used to further adjust the final regression models that also controlled for age, race, income, metastases, and mortality risk (as calculated by the NIS using the DRGs). The selection of variables in the final regression models were determined by the clinically and/or statistically significant variables identified in bivariable analysis and/or the existing literature, with selection also augmented by forward and backward stepwise regression. To optimize the likelihood of robust, validated, and replicable results, the performance of the final regression models was first assessed by backward propagation neural network machine learning by accuracy and root mean squared error (RMSE) to ensure they were comparable based on an integrated hybrid methodology of traditional statistics reinforced by machine learning. This novel methodology has been previously demonstrated as causal inference results (that are more familiar to medical science audiences) which can be confirmed and replicated automatically through machine learning (which is capable of handling greater data volume, speed, and complexity and so may accelerate future real-time findings on larger high-dimensional datasets as they already increasingly do for other economic sectors outside of medicine), all while generating more accurate and prompt results compared to traditional statistics alone [14–17]. The following diagnostics were conducted also to optimize performance of the final regression models: Hosmer-Lemeshow’s goodness-of-fit test, Akaike’s and Schwarz’s Bayesian information criteria, correlation matrix, AUC, tolerance, variance inflation factor, specification error, and multicollinearity. 95% confidence intervals (CIs) were reported for the final regression results with statistical significance determined by a two-tailed *p*-value of < 0.05. This has been demonstrated in recent studies. STATA 14.2 (STATACorp, College Station, Texas, USA) was used for statistical analysis, and Java 9 (Oracle, Redwood Chores, California, USA) was used for machine learning analysis.

## Results

### Descriptive statistics & bivariable analysis

Of the 30,195,722 total hospital admissions in 2016 within the United States, 39,254 (0.13%) underwent TAVR and 69,450 (0.23%) underwent SAVR, and 661,286 (2.19%) died among all hospitalized adults nationally in 2016. Among patients with cancer (Table 1), TAVR patients compared to both MM and SAVR patients were more likely to be older and have non-private insurance, diabetes, hypertension, congestive heart failure, and chronic kidney disease stage 3–5 (all *p* < 0.001). TAVR versus MM and SAVR patients had the lowest mortality (respectively, 1.43% versus 3.99% versus 3.24%, all *p* < 0.001) and median length of stay in days (respectively, 4.59 [standard deviation {SD} 4.56] versus 5.45 [6.34] versus 9.10 [7.09], all *p <* 0.001).

**Table 1:** Descriptive statistics among all adult hospitalizations and bivariable analysis by aortic stenosis treatment modality among cancer patients^a^

Variables	Sample	Treatment			*P*-value	
	*N* = 30,195,722	MM (*n* = 4,640,563 [99.60%])	TAVR (*n* = 9318 [0.20%])	SAVR (*n* = 8852 [0.19%])	TAVR versus MM	TAVR vs SAVR
Demographics, no. (%)						
Age, mean (SD)	68.70 (14.30)	68.67 (14.31)	81.00 (7.93)	71.52 (9.35)	**< 0.001**	**< 0.001**
Female	15,254,879 (50.52)	(50.54)	(40.2)	(32.61)	**< 0.001**	**< 0.001**
Race, nonwhite	7,132,230 (23.62)	(23.65)	(10.00)	(10.97)	**< 0.001**	0.349
Insurance, non-private	23,380,548 (77.43)	(77.4)	(93.23)	(79.23)	**< 0.001**	**< 0.001**
Medical history						
Diabetes	6247,495 (20.69)	(20.68)	(26.02)	(20.20)	**< 0.001**	**< 0.001**
Hypertension	19,790,276 (65.54)	(65.49)	(88.13)	(81.14)	**< 0.001**	**< 0.001**
Hyperlipidemia	11,869,938 (39.31)	(39.25)	(69.29)	(70.20)	**< 0.001**	0.546
Congestive heart failure	1,926,487 (6.38)	(6.33)	(34.3)	(12.95)	**< 0.001**	**< 0.001**
Smoking	332,153 (1.10)	(1.10)	(0.37)	(1.17)	**< 0.001**	**0.005**
Depression	3,895,248 (12.90)	(12.91)	(7.6)	(9.43)	**< 0.001**	**0.046**
Cirrhosis	742,815 (2.46)	(2.47)	(1.53)	(0.56)	**0.009**	**0.004**
CKD 3–5	4,055,285 (13.43)	(13.4)	(24.54)	(11.55)	**< 0.001**	**< 0.001**
Outcomes, median (range)						
Mortality, no. (%)	1,201,790 (3.98)	(3.99)	(1.43)	(3.24)	**< 0.001**	**< 0.001**
Length of stay, days	5.46 (6.34)	5.45 (6.34)	4.59 (4.56)	9.10 (7.09)	**< 0.001**	**< 0.001**
Cost, dollars	60,612.22 (87,774.68)	60,002.30 (86,913.86)	210,106.90 (110,707.70)	219,202.30 (179,519.90)	**< 0.001**	0.065

^a^*MM* medical management, *TAVR* transcatheter aortic valve replacement, *SAVR* surgical aortic valve replacement, bold = statistically significant, *CKD* chronic kidney disease

Among TAVR subjects, 23.53% were done in patients with cancer and 0.67% in those with metastatic cancer. In sub-group analysis among patients 65 years of age and older and elevated mortality risk (moderate, major, and extreme but not minor as calculated by the NIS according to DRGs), still significantly fewer TAVR procedures were done in patients with cancer versus without it (23.82% versus 76.18%, *p* < 0.001). Yet mortality was comparable among patients with and without cancer who underwent TAVR (1.43% versus 1.98%, *p* = 0.118), including in the similar above sub-group of patients of comparable age and mortality risk (1.57% versus 1.96%, *p* = 0.297). The leading primary malignancies in which TAVR was done included: prostate (23.06%), skin (16.71%), breast (15.34%), bladder (7.88%), colon (6.45%), with the leading malignancies among SAVR subjects being similar including prostate (26.69%), skin (21.77%), breast (13.32%), bladder (5.54%), and colon (5.15%).

Among TAVR performing hospitals, the mean number of procedures annually was 29.94 (SD 21.00) with the tertiles being 1–17, 18–36, and 37–112. In sub-group analysis among patients with cancer, descriptive statistics and bivariable analysis by aortic stenosis treatment modality are provided in Table 1.

### TAVR versus MM

TAVR versus MM patients were significantly older (80.28 years [SD 8.32] versus 57.46 [SD 20.34; *p* < 0.001), and less likely to be female (45.78% versus 58.22%, *p* < 0.001), non-white (13.53% versus 32.26%, *p* < 0.001), and die inpatient (1.85% versus 2.19%, *p* = 0.039), while being more likely to have a longer LOS (mean 5.16 days [SD 6.04] versus 4.72 [SD 6.33], *p* < 0.001) and higher cost (mean USD $216,458.70 [SD 136,223.5] versus $49,903.50 [81,963.57], *p* < 0.001).

In fully adjusted analysis for all hospitalized adults, TAVR compared to MM significantly reduced mortality (OR 0.52, 95%CI 0.43–0.63; *p <* 0.001) and non-home discharge (OR 0.84, 95%CI 0.84–0.85; *p <* 0.001) overall. While there was non-significantly reduced mortality for patients with cancer versus without (OR 0.71, 95%CI 0.45–1.11; *p* = 0.133) and more so for patients with metastatic disease (OR 0.29, 95%CI 0.04–2.20; *p* = 0.233), there was a significant reduction among patients with cancer in the LOS (beta days − 0.72, 95%CI -1.04- -0.40; *p* < 0.001) and cost (beta USD $-5186, 95% CI -8627.01- -1745.08; *p* = 0.003) (Table 2). In stratified analysis, there was no significant association with mortality between TAVR versus MM for patients with cancer with active versus prior cancer, solid versus non-solid malignancies, thrombocytopenia versus not, or radiation versus non-radiation.

**Table 2:** Propensity score adjusted multivariable regression of outcomes by aortic valve replacement versus medical management for cancer and non-cancer patients^a^

Variable	OR (95%CI; *p*-value)					
	Mortality		Cost (United States Dollars)		Length of stay (Days)	
Age by 10 years	TAVR vs MM	TAVR vs SAVR	TAVR vs MM	TAVR vs SAVR	TAVR vs MM	TAVR vs SAVR
Non-white race	**1.01 (1.01–1.01)**	1.00 (1.00–1.01)	**−43.90 (−47.04- -40.76)**	−3.66 (− 192.18–184.84)	**− 0.02 (− 0.02- -0.02)**	**−0.06 (− 0.07- -0.05)**
Zip code income	**1.03 (1.02–1.04)**	0.98 (0.78–1.23)	**3953.27 (3833.21-4073.34)**	**31,486.94 (25,653.33-37,320.55)**	**0.31 (0.30–0.32)**	**1.12 (0.84–1.40)**
1st quartile	Reference	Reference	Reference	Reference	Reference	Reference
2nd quartile	**0.96 (0.95–0.98)**	0.91 (0.71–1.16)	**2120.83 (1976.57-2265.09)**	4416.36 (− 1564.70-10,397.41)	**− 0.13 (− 0.15- -0.12)**	−0.20 (− 0.49–0.09)
3rd quartile	**0.92 (0.90–0.93)**	0.98 (0.77–1.25)	**5372.67 (5224.71-5520.64)**	**16,506.95 (10,558.02-22,455.89)**	**−0.17 (− 0.18- -0.16)**	**−0.49 (− 0.78- -0.20)**
4th quartile	**0.93 (0.91–0.95)**	0.92 (0.71–1.19)	**9909.08 (9751.90-10,066.26)**	**31,220.53 (25,154.54-37,246.52)**	**−0.11 (− 0.12- -0.09)**	−0.25 (− 0.54–0.04)
Procedure	**0.52 (0.43–0.63)**	0.90 (0.70–1.16)	**164,413 (162,704.90-166,121.10)**	**52,098.41 (46,359.88-57,836.93)**	−0.01 (− 0.17–0.14)	**− 3.85 (− 4.13- -3.58)**
Malignancy	**1.08 (1.06–1.10)**	1.07 (0.78–1.47)	**2606.99 (2441.96-2772.02)**	− 7911.60 (− 15,835.76–12.55)	**0.06 (0.05–0.08)**	**− 0.89 (− 1.27- -0.51)**
Metastases	**1.95 (1.91–2.00)**	0.63 (0.15–2.70)	**− 6706.27 (− 7033.93- -6378.60)**	922.71 (− 575.25–22,025.82)	**− 0.31 (− 0.34- -0.28)**	0.86 (− 0.81–2.54)
With Procedure	0.71 (0.45–1.11)	0.76 (0.43–1.32)	**−5186.04 (− 8627.01- -1745.08)**	10,725.28 (− 575.25–22,025.82)	**− 0.72 (− 1.04- -0.40)**	0.35 (− 0.20–0.89)
Mortality risk by DRG	**7.88 (7.81–7.96)**	10.74 (8.93–12.93)	**8108.62 (8042.55-8174.68)**	**21,602.97 (18,898.19-24,307.74)**	**2.10 (2.09–2.10)**	**4.29 (4.18–4.41)**
Length of stay, days			**7514.37 (7505.36-7523.38)**	**14,544.34 (14,262.47-14,826.21)**		

^a^*TAVR* transcatheter aortic valve replacement, *MM* medical management, *SAVR* surgical aortic valve replacement, *DRG* Disease Related Group, bold = statistically significant

**Fig. 1:**
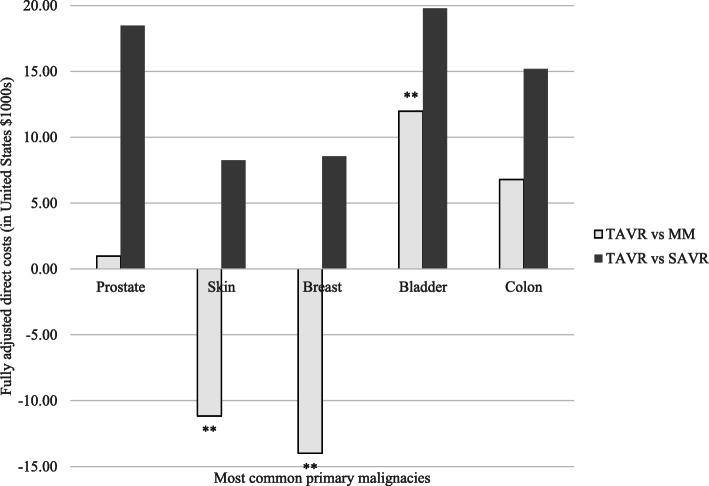
Propensity score adjusted multivariable regression of outcomes by aortic valve replacement versus medical management by primary cancer

Assessing primary malignancies separately, there was no significant association with mortality between TAVR versus MM for patients with cancer with the top five above primary malignancies. TAVR versus MM did significantly reduce costs for those with skin cancer (beta $-11,175.68, 95%CI -18,134.07- -4217.30; *p* = 0.002) and breast (beta $-14,012.76, 95%CI -21,054.98- -6970.53; *p* < 0.001) while significantly raising it for those with bladder cancer (beta $11,974.51, 95% CI 1545.01-22,404.01; *p* = 0.024) (Fig. 1).

### TAVR versus SAVR

TAVR versus SAVR patients were significantly older (mean 80.28 years [SD 8.32] versus 66.40 [SD 12.62], *p* < 0.001) and more likely to be female (45.78% versus 33.05%, *p <* 0.001) and less likely to be non-white (13.53% versus 17.58%, *p* < 0.001), die (1.85% versus 3.31%, *p <* 0.001), and be discharged non-home (44.93% versus 64.84%, *p <* 0.001). TAVR versus SAVR patients also had lower LOS (mean 5.16 [SD 6.04] versus 10.19 [SD 9.51], *p <* 0.001) and cost (mean USD $216,458.70 [SD 136,223.5] versus $242,302.10 [SD 232,242.80], *p <* 0.001).

TAVR versus SAVR for patients with cancer non-significantly decreased mortality (OR 0.76, 95%CI 0.43–1.32; *p* = 0.326) and non-home discharge (OR 0.89, 95%CI 0.76–1.05; *p* = 0.172), but increased LOS (beta 0.35, 95%CI -0.20-0.89, *p* = 0.214) and costs (beta USD $10,725.28, − 575.25-22,025.82; *p* = 0.063). There was no significant association with mortality, LOS, nor cost between TAVR versus SAVR for patients with cancer with the top five above primary malignancies. In stratified analysis, there was no significant association with mortality between TAVR versus SAVR for patients with cancer with active versus prior cancer, solid versus non-solid malignancies, thrombocytopenia versus not, or radiation versus non-radiation.

### TAVR procedural volume

TAVR procedural volume did not significantly impact mortality or LOS, but compared to the lowest tertile, the second tertile significantly decreased costs (beta $-10,498.49, 95%CI -16,575.01- -4421.97; *p* = 0.001) while the third tertile significantly increased costs (beta $11,742.95, 95%CI 5455.22-18,030.68; *p <* 0.001).

Among all TAVR subjects, there were no significant race or geographic disparities for mortality; there were significantly different LOS by geographic region relative to New England: West North Central (beta − 1.26, − 1.95- -0.57; *p* < 0.001), Mountain (beta − 1.07, 95%CI -1.79- -0.35; *p* = 0.004), and Pacific (beta − 0.72, 95%CI -1.31- -0.14; *p* = 0.016). All regions had significantly increased costs relative to New England with the most expensive regions being the Pacific (beta $120,651.20, 95%CI 108,823.80-132,478.50; *p* < 0.001), Mountain (beta $91,538.29, 95%CI 77,089.44-105,987.10; *p* < 0.001), and Mid-Atlantic (beta $86,865.73, 95%CI 75,686.65-98,044.80; *p* < 0.001). Among TAVR subjects who also have cancer, there were no significant race or geographic disparities for mortality; all geographic regions had significantly lower LOS relative to New England with the lowest LOS being for Mountain (beta − 2.19, 95%CI -3.38- -1.01; *p* < 0.001), West North Central (beta − 2.01, 95%CI -3.14- -0.89; *p* < 0.001), and West South Central (beta − 2.00, 95%CI -3.05- -0.94; *p* < 0.001). Yet all regions had significantly greater costs relative to New England with the greatest being: Pacific (beta $120,414.30, 95%CI 99,695.70–141.132.80; *p <* 0.001), Mid Atlantic (beta $94,020.50, 95%CI 74,543.15-113,497.80; *p <* 0.001), and Mountain (beta $80,620.67, 95%CI 51,101.14-107,140.20; *p <* 0.001).

## Discussion

This is the first known nationally representative analysis of mortality and cost for patients with versus without cancer (including by primary cancer) by TAVR versus MM and TAVR versus SAVR, including the first to use a comprehensive machine learning-augmented propensity score analysis integrating traditional statistics and machine learning [13–16]. It provides novel multi-center evidence that TAVR is preferentially performed less often in patients with cancer versus without it despite the above groups having seemingly comparable risk profiles and mortality without significantly increased costs. TAVR when compared with MM in patients with cancer produced comparable outcomes; however, TAVR patients had shorter lengths of stay and incurred less cost. Assessment of quality of life for patients with cancer who underwent TAVR could not be assessed but would not be expected to be at significant variance compared to patients without cancer. Additionally, TAVR may have significant geographic disparities in both LOS and cost without mortality or racial disparities among patients with cancer suggesting such patients do not have increased peri-procedural risk which should limit their access to this treatment (though this study could not assess adequately if minorities and rural patients undergo TAVR at lower rates because of under-diagnosis of which previous research has raised the concern) [2]. The analysis further supports that these cost differences may be driven at least in part by particular primary malignancies, such as TAVR versus MM potentially reducing hospital costs for those with breast and skin cancers without significant differences by primary cancer for TAVR versus SAVR.

This study thus provides novel, robust evidence that suggests TAVR’s clinical and cost benefit should be extended to patients with cancer. This may provide the benefit not only of treating AS to reduce CVD morbidity and mortality, but also cancer outcomes by improving their functional status and thus eligibility to receive cancer therapeutics which previously may have been withheld from them due to hemodynamically or functionally compromising AS. The study additionally provides the first known granular analysis by primary cancer type and TAVR versus MM and TAVR versus SAVR, allowing greater insights how cost may affect pre-procedure management of such patients. Of course this study cannot support the unfounded assertion that TAVR or SAVR should be extended to all patients with cancer, but it may cautiously support the hypotheses that such interventions may (a) be underutilized among patients with cancer unnecessarily, and (b) yet provide net benefit which thus prompts more personalized consideration of offering such patients these interventions when clinically indicated despite their cancer status.

Prior studies suggest there may be increased mortality in TAVR for adults under 80 compared to older patients, and for those undergoing TAVR versus SAVR [18]. Additionally, patients undergoing TAVR versus SAVR are older and have a greater number of comorbidities [19, 20]. This study thus adds to such literature the novel findings that TAVR versus MM may reduce LOS and cost but not mortality for patients with cancer, and that TAVR versus SAVR may have comparable outcomes for this patient group. Furthermore, this is the first known NIS study of patients undergoing TAVR that shows there are geographic disparities in LOS and cost among adult hospitalized patients overall and those specifically with cancer, while suggesting there is no improvement in outcomes by centers with increased procedural volume of TAVR or SAVR.

The strengths of this study include its novel utilization of a nationally representative multi-center dataset to analyze among all adult hospitalized patients TAVR versus MM and specifically TAVR versus SAVR, which was a comprehensive analysis of multiple outcomes including a sub-group disparity analysis, and utilized a robust propensity score methodology augmented by a machine learning analysis (with implications for later more sophisticated and automated machine learning-based analysis with increasing amounts of data). The above analysis is thus novel even among other NIS studies by increasing the external validity and thus generalizability of the study by analyzing all adult hospitalizations to more finely hypothesize about the true association between TAVR and alternatives and clinical outcomes for patients with cancer, while using a robust approach to optimize internal validity with a well-accepted study design that reduces bias and type I and II error through its large multi-center dataset and propensity score analysis.

The above strengths were utilized in an attempt to ameliorate as much as possible the substantive limitations of this study which include its non-randomized study design and administrative data limited to short-term outcomes without severity grading of AS or comprehensively detailed grading of surgical risk. This limitation may be particularly notable in two ways that may limit external validity. (1) Patients undergoing MM may have moderate or severe AS (but not yet symptomatic severe cases) in which their providers are monitoring but not yet opting for interventional management due to inadequate suspected net benefit. If anything this approach would favor improved outcomes in the MM group which was not consistently demonstrated in this study. (3) This study does suggest comparable mortality by TAVR versus SAVR for patients with prostate, skin, breast, bladder, and colon cancer; these patients may have superior clinical outcomes in terms of their cancer (holding constant other health aspects including cardiovascular and valvular disease) compared to patients with other cancer types or lack thereof and so they may be preferentially selected for TAVR and/or SAVR versus MM and thus overrepresent cancer patients who have good post-interventional AS treatment.

The above analysis sought to reduce the impact of such potential bias by a robust analytic method which included controlling for the severity of clinical illness and other such factors detailed in the methods and results which may impact not only the likelihood of undergoing TAVR or SAVR but also suffering inpatient mortality to thus as best as possible assess the independent association among the AS treatments (TAVR versus SAVR versus MM) and cancer status. Further, the study demonstrates the lower TAVR prevalence among patients with cancer despite matching for age and mortality risk (and despite prior research demonstrating their similar co-prevalence of severe AS relative to age-matched patients without cancer and their historic exclusion from valvular trials) [12, 21], which supports the hypothesis that patients with cancer may receive only MM despite having severe AS and overall clinical severity profiles amenable to TAVR or SAVR (but are not treated as such due to provider hesitation with their cancer status). These considerations from the limitations nonetheless underline the potential importance of this study advancing this novel topic for such often overlooked patients to hopefully more comprehensively and with more confidence investigate such questions with a large dataset and robust multi-faceted analytic techniques, and so hopefully prepare the way for future longer-term and randomized studies including for patients with more detailed AS status and other cancer types. Future studies are required to confirm and expand these results including by primary cancer type to ensure the best available data informs treatments for cardio-oncology patients to produce the best possible outcomes for them.

## Conclusions

This is the first known nationally representative comprehensive analysis of inpatient mortality and cost by cancer status (including primary cancer type) and AS treatment modality (TAVR, SAVR, and MM). It utilizes a robust and novel traditional statistical approach of causal inference supported by machine learning to suggest the unique finding that TAVR’s clinical, cost, and racially equitable advantages may be safely extended to patients with cancer with not only cardiovascular net benefit (treating the primary problem of AS) but also oncological benefit (improving their functional status to potentially allow expanded cancer treatments).

## Data Availability

The data is available by purchase through the United States Agency for Healthcare Research and Quality (AHRQ) Healthcare Cost and Authorization Project (HCUP) Central Distributor (hcup-us.ahrq.gov).
